# Case report: Child chronic nonbacterial osteomyelitis with rapid progressive scoliosis-an association with disease?

**DOI:** 10.3389/fped.2023.1076443

**Published:** 2023-03-21

**Authors:** Xiaojun Shi, Xiujuan Hou, Haiqin Hua, Xia Dong, Xiaoping Liu, Fengjiao Cao, Chen Li

**Affiliations:** ^1^ Department of Rheumatology, Dongfang Hospital, Beijing University of Chinese Medicine, Beijing, China; ^2^ Department of Radiology, Dongfang Hospital, Beijing University of Chinese Medicine, Beijing, China; ^3^ Department of Rheumatology, Fangshan Hospital, Beijing University of Chinese Medicine, Beijing, China

**Keywords:** scoliosis, children, autoimmune disease, rapid progress, chronic nonbacterial osteomyelitis, Adalimumab, zoledronic acid

## Abstract

**Background:**

Chronic nonbacterial osteomyelitis (CNO) is an auto-inflammatory bone disease that usually develops in childhood. Spinal involvement is a common manifestation of CNO, but it is rare for CNO to lead to rapid progression of scoliosis deformity. Here we present a 9-year-old girl with acute scoliosis with CNO and scoliosis progressed rapidly in 2 months.

**Case Presentation:**

A 9-year-old girl presented bilateral shoulder inequality with pain in the left hypochondrium for 2 months. Standing spinal x-rays showed right convex scoliosis with a 25° Cobb angle. Chest magnetic resonance imaging (MRI) showed that the T8 vertebra was flattened and local bone was destroyed with bone marrow edema. The bone biopsy showed evidence of fibrosis and chronic inflammatory changes with no specific diagnosis. One month later, her scoliosis and bone destruction deteriorated obviously. Thoracic vertebra MRI showed that the T8 vertebra had a compression fracture. ^99m^Tc-MDP whole-body bone scintigraphy showed intense uptake at T8/9 and the right sacroiliac joint. She was diagnosed with CNO accompanied by rapidly progressive scoliosis. The scoliosis was successfully treated with adalimumab and zoledronic acid, which showed significant improvement after 6 months of follow-up.

**Conclusion:**

Zoledronic acid and adalimumab successfully treated CNO with rapidly progressive scoliosis, but could not prevent vertebral compression.

## Introduction

Chronic nonbacterial osteomyelitis (CNO) is a rare aseptic and chronic auto-inflammatory bone disease that usually occurs in childhood (1, 2). Clinical manifestations vary in severity from unifocal to multifocal, with chronic recurrent multifocal osteomyelitis (CRMO) being a severe form of CNO (3). The incidence of CNO is unknown, with some surveys suggesting an estimated annual incidence of 4/1,000,000, but the incidence may be grossly underestimated due to the lack of authoritative classification criteria and delay in diagnosis (4). CNO usually presents as insidious bone pain with or without systemic features (4). It commonly affects the metaphyses of long bone, followed by the spine, clavicle and mandible (5, 6). CNO scoliosis is relatively rare and no cases of rapid scoliosis progression have been reported. Here we report a 9-year-old girl with acute scoliosis secondary to CNO whose scoliosis progressed rapidly within two months and was successfully treated with adalimumab and zoledronic acid.

## Case report

**Figure 1:**
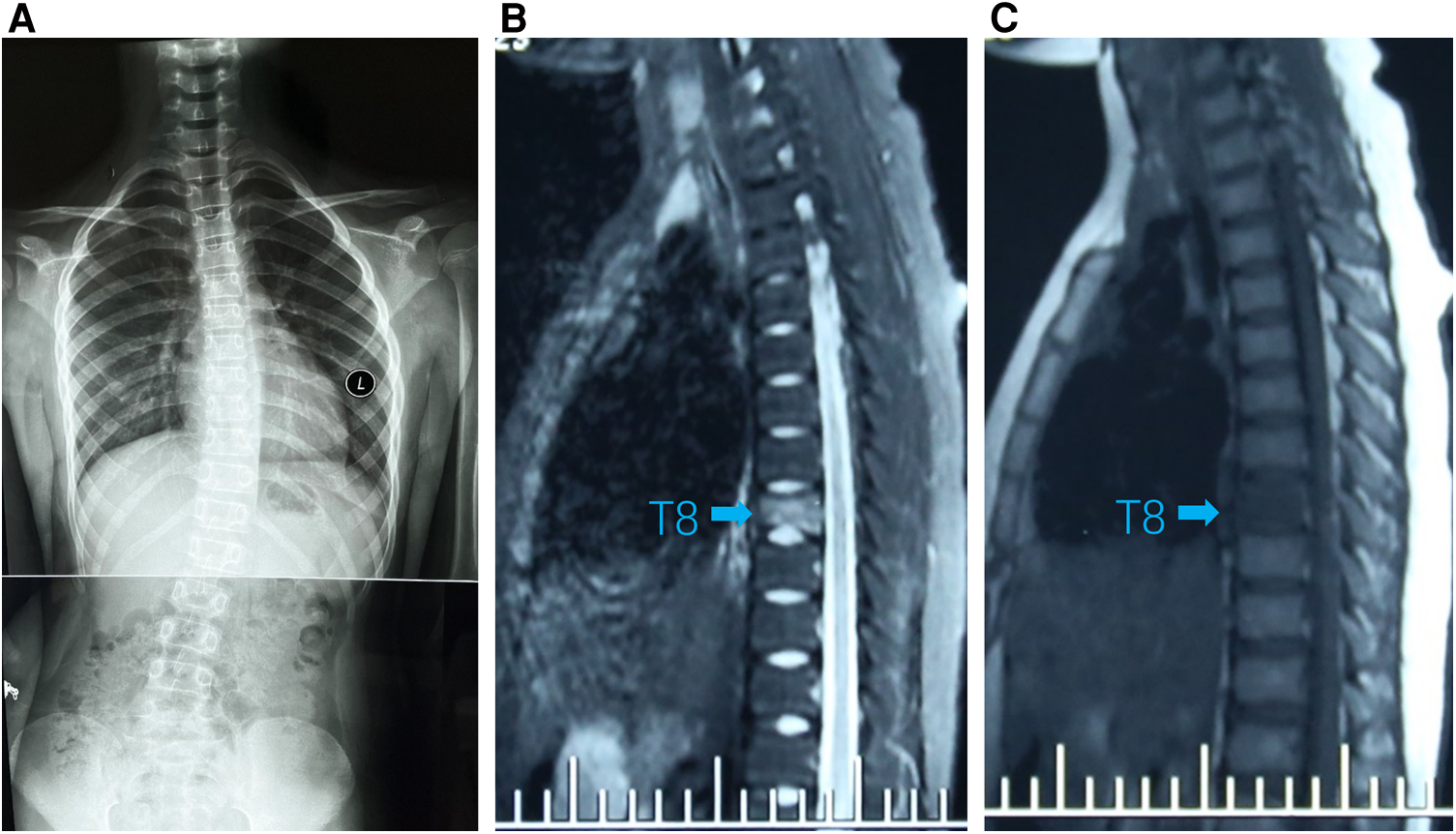
(**A**) Standing spinal x-rays showed right convex scoliosis. (**B**) T1-weighted MRI image showed that the T8 vertebra was flattened and local bone was destroyed (arrow). (**C**) T2-weighted MRI image showed bone marrow edema in T8 vertebra (arrow).

**Figure 2:**
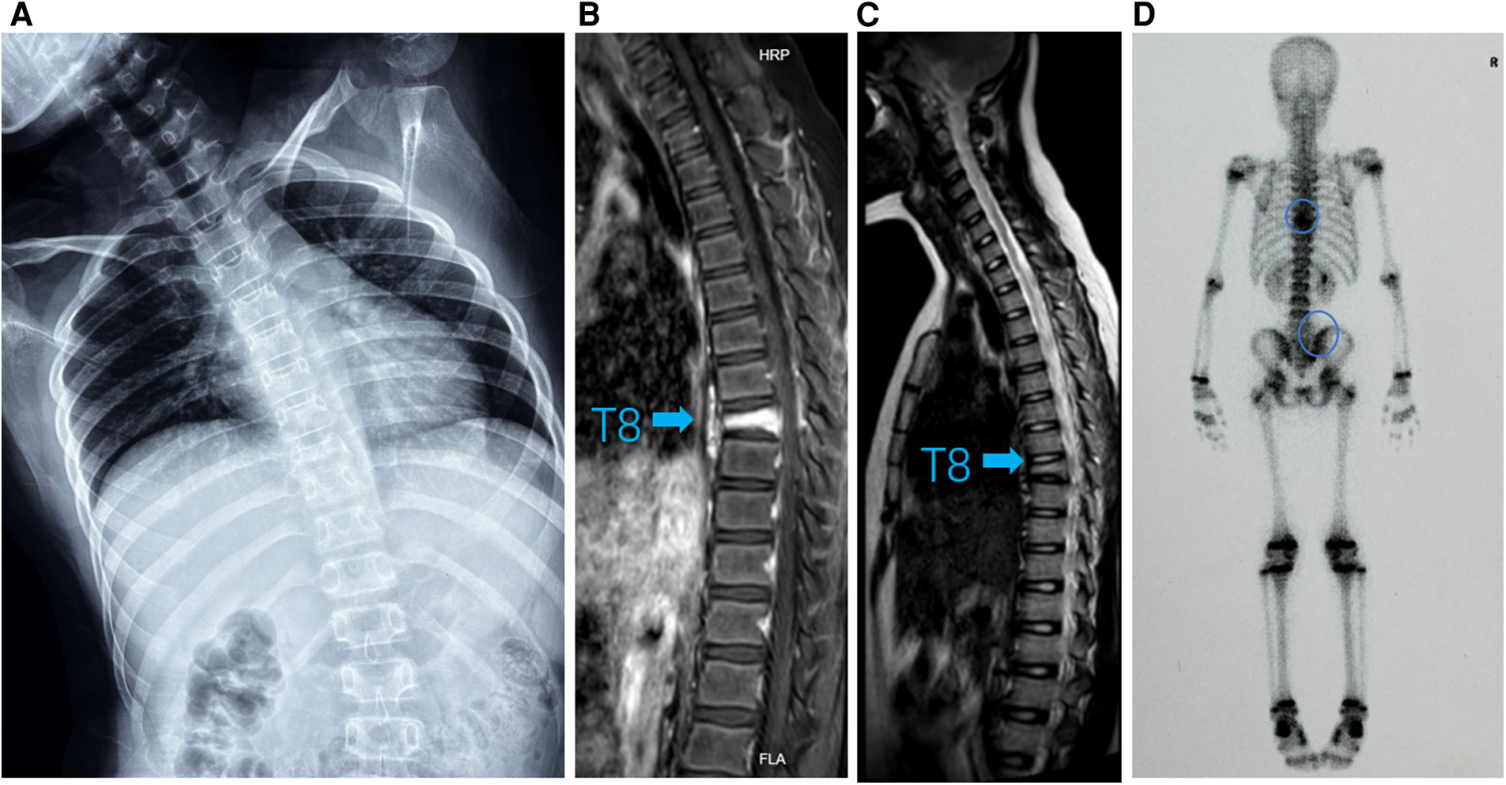
(**A**) Standing spinal x-rays showed heavier right scoliosis. (**B**) T1-weighted MRI image and (**C**) T2-weighted MRI image of the thoracic vertebra showed that the T8 vertebra had a compression fracture (arrow). (**D**) ^99m^Tc-MDP whole-body bone scintigraphy showing hot spots in T8/9 and right sacroiliac joint with posterior view (circle).

A 9-year-old girl presented to the clinic with bilateral shoulder inequality and left hypochondrium pain for 2 months. She had no history of trauma, serious medical problems, or family history of skeletal problems or psoriasis. She developed left hypochondriac pain on 9 June 2022, and two weeks later, her parents found her back curved to the right with skin lesions on both sides of the left lower extremity. The lesions on the inner calf presented as four green bean-sized pustules that partially ruptured, and on the outer side as an oval red squamous patch. Standing spinal x-rays showed a right convex scoliosis with a 25° measured by the Cobb angle method. Chest MRI showed that the T8 vertebra was flattened and local bone was destroyed with bone marrow edema (Figure 1). We biopsied her thoracic vertebrae and skin lesions. She then underwent pathological biopsies of the T8 vertebra and lower limb skin lesions (Supplementary Figure S1). The bone biopsy showed evidence of fibrosis and chronic inflammatory changes with no specific diagnosis. The skin biopsy showed chronic inflammation. Immunohistochemistry results revealed CD207(-), CD1a (−), CD68(focal +), S-100 (−), CD163 (−). One month later, her scoliosis and bone destruction deteriorated obviously, and back pain occurred. MRI of the thoracic vertebra showed that the T8 vertebra had a severe compression fracture (Figure 2A–C). The right sacroiliac joint and T8/9 regions were shown to have intense absorption by ^99m^Tc-MDP whole-body bone scintigraphy (Figure 2D).

Physical examinations showed normal vital signs and the shoulders were imbalanced, the left shoulder was higher than the right shoulder, and the spinous process of the back was complete to the right. Laboratory assays revealed a slight elevation of C-reactive protein (CRP, 8.41 mg/L, normal range 0–8.0 mg/L) and rheumatoid factor test was negative. Combined with medical history and auxiliary examination, we diagnosed the patient with CNO accomplished by scoliosis.

**Figure 3:**
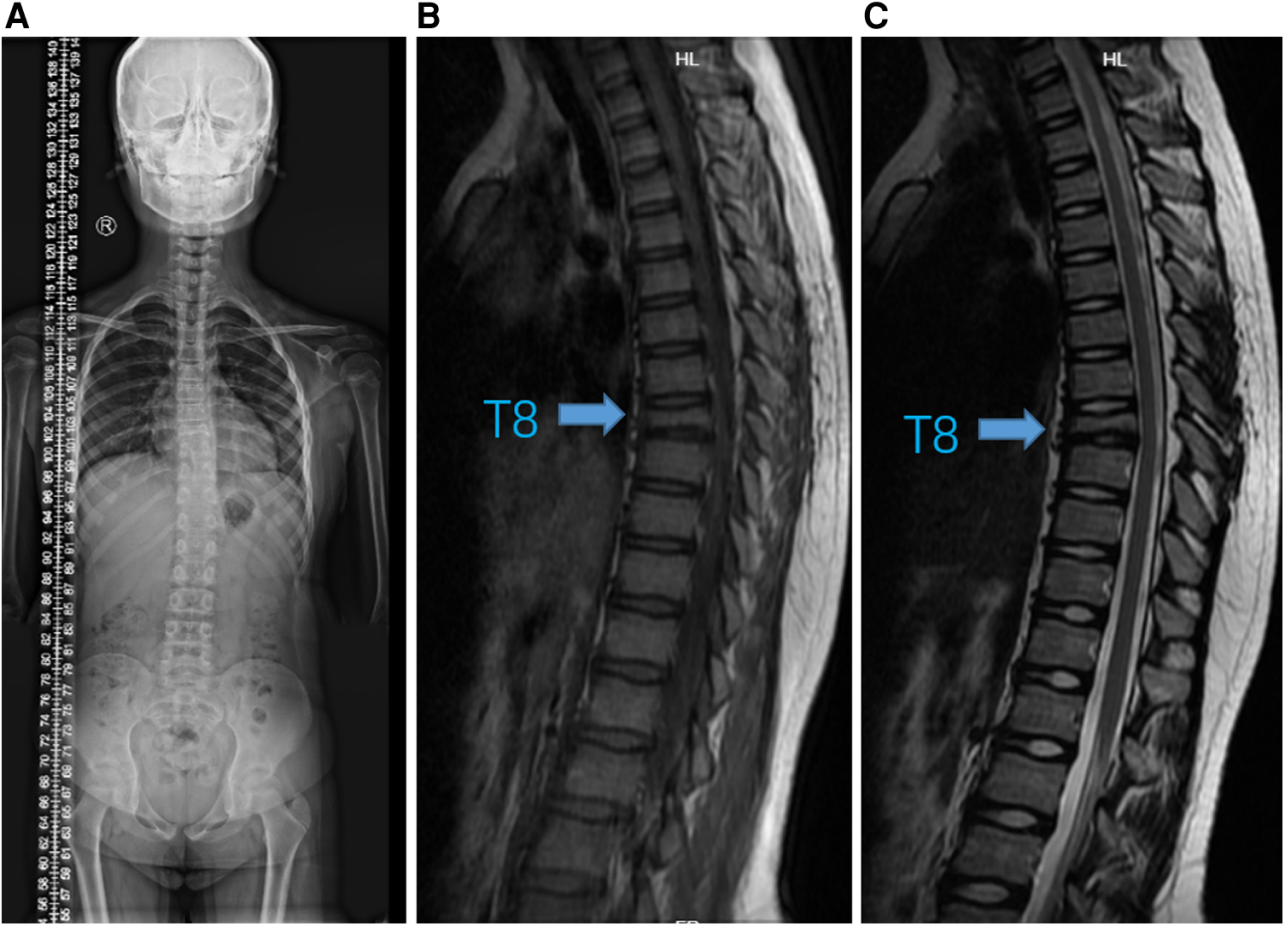
Imaging changes after six months of therapy. (**A**) Standing spinal x-ray. (**B**) T1-weighted and (**C**) T2-weighted MRI images showed that T8 vertebra remained stable without progression (arrow).

Immediately following, the girl has been treated with adalimumab (40 mg once every two weeks) and zoledronic acid (2.5 mg once every three weeks) for six months. We followed up at 3 and 6 months, and the patient's CRP had returned to normal, routine blood and biochemical tests were still negative. The standing spine x-ray and MRI of the thoracic spine (Figure 3 and Supplementary Figure S2) were re-examined and showed significant improvement from before. Meanwhile, we extracted peripheral blood genomic DNA from the patient and her parents and performed whole-exome sequencing, which revealed a variation in the *LAMB3* gene.

After treatment, the girl showed significant improvement in scoliosis, reduction in bone marrow edema and back pain, and improvement in skin lesions (Supplementary Figure S3), although vertebral collapse remained with no significant change.

## Discussion

CNO has been characterized for at least 50 years since it was first described by Gideon et al. in 1972 (7). However, there is a lack of internationally accepted diagnostic criteria for CNO, and the classification and diagnostic scores proposed by Jansson et al. are currently the more commonly used criteria (8). As an exclusionary diagnosis, CNO must be distinguished from tumors, infectious osteomyelitis and langerhans cell histiocytosis (LCH), et al. LCH, similar to CNO, can present with osteolytic changes in the vertebra, but the pathology is seen with an abnormal infiltration of Langerhans histiocytes and immunohistochemistry of CD1a/CD207 (+) (9). This girl had no fever and no abnormal blood counts, while bone biopsy and pathology revealed no tumor cells, langerhans cells or bacterial infection. The final diagnosis was CNO with scoliosis, fulfilling the diagnostic criteria for CNO proposed by Jansson et al. (8).

Early reports of spinal involvement in CNO/CRMO were considered rare in the literature, but in the last 20 years there has been a gradual increase with an incidence of approximately 10%–38% (3, 10–13). We reviewed the vertebral involvement of CNO/CRMO patients in the literature since 2000 (Table 1). A PRISMA flow chart of the literature screening process is in the Supplementary Material (Supplementary Figure S4). The relationship between SAPHO and CNO/CRMO in children is unclear, so SAPHO cases were not included (24). We found that the thoracic vertebra were the most commonly affected vertebra, followed by the lumbar, cervical and sacral vertebra, which is similar to the data summarized by S. E. Anderson et al. before 2000 (23). Major vertebral involvement included osteolytic changes (including collapse, compression fractures and complete destruction), kyphosis, scoliosis and bone marrow edema. Scoliosis deformities were seen in 2 studies (16, 20), one of which showed acute scoliosis deformity similar to ours. In contrast to our case, which showed a rapid deterioration within 2 months, his case was very stable, with no significant progression of the scoliosis (20).

**Table 1:** Review of CNO/CRMO with spinal lesion.

First author-year	Number of cases	Type of spine lesions	Site of spin involved	Treatment
Sirisha Koneru-2022 (14)	1	bone marrow edema	T8, L1	naproxen
Dheeraj Batheja-2021 (15)	1	Osteolytic lesion	T6–8	bisphosphonates and NSAIDs
Kazuta Yamashita-2021 (16)	1	Osteolytic lesion and bone marrow edema	T4, T7, T11	NSAIDs and pamidronate
C Galeotti-2015 (17)	1	Osteolytic lesion and kyphotic	T5–6	NA
Cheng William Hong-2015 (18)	1	bone marrow edema	T6, T8, S1	alendronate
Shabina Habibi-2013 (19)	1	Osteolytic lesion	NA	pamidronate
Kedar Deogaonkar-2008 (20)	1	Osteolytic lesion and scoliosis	T10, L2–3	NSAIDs
Tony Walls-2006 (21)	1	Osteolytic lesion	T6–7	Ibuprofen
Colleen S Y Chun-2004 (22)	1	Osteolytic lesion	S1	prednisone, alendronate, and naprosyn
S E Anderson-2003 (23)	3	Osteolytic lesion and kyphotic	P1: T4, T6, T8	NA
		Osteolytic lesion	P2: C2–3, T9	NA
		Osteolytic lesion	P3: L3	NA

In addition to their anti-inflammatory and analgesic effects, bisphosphonates can reduce the development of osteoclast precursor cells and promote the apoptosis of mature osteoclasts (25). So we treated her with zoledronic acid. After 6 months of treatment, the scoliosis had largely improved, but the spinal collapse persisted. In severe spinal involvement, pamidronate may be more effective than zoledronic acid, encouraging bone healing and preventing progression of vertebral compression (10, 26–28). In patients with spinal deformity, the use of a plaster corset to support the spine may also be beneficial (10).

Imbalanced anti-inflammatory and pro-inflammatory pathways are important molecular mechanisms involved in developing CNO. Studies have shown that serum pro-inflammatory molecules (IL-6, TNF-α, IL-1β) are increased and anti-inflammatory factors (IL-10, IL-19) are decreased in CNO patients (29). The imbalance between pro- and anti-inflammatory cytokines can be restored by TNF-α inhibitors (TNFi) which are recommended as the preferred second-line treatment for patients with associated inflammatory skin lesions (30). Our patient had pustular lesions on the lower limbs, so we added the TNFi adalimumab. In 2019, the Childhood Arthritis and Rheumatology Research Alliance has developed three consensus treatment plans for patients with NSAID-refractory CNO, including bisphosphonates and TNFi (31).

In this patient, the scoliosis deformity developed rapidly over a 2-month period and resolved after treatment. The images showed a parallel progression of thoracic spine destruction and scoliosis. It is likely that the spinal destruction and pain from CNO were responsible for the progression of the scoliosis.

There is evidence that CNO may be caused by genetic factors (3, 32). Our preliminary whole exome sequencing results suggest this patient has a *LAMB3* (c.595G > A) gene variant which is a protein-coding gene in exon7. The product encoded by *LAMB3* is laminin beta3 that belongs to a family of basement membrane proteins. Laminin beta3 is a unique component of laminin 332, which is a novel negative regulator of osteoclastogenesis in the bone microenvironment and has an important role in the control of normal bone remodeling (33). We recommend further functional studies to elucidate its pathogenic impact.

In conclusion, we report a case of CNO with rapid scoliosis in a patient who had significant relief of scoliosis after treatment with zoledronic acid and adalimumab, but was failed to avoid vertebral compression. We need to properly identify the spinal involvement of CNO, which can progress rapidly in combination with scoliosis, and develop an individuation therapy.

## Data Availability

The original contributions presented in the study are included in the article/**Supplementary Material**, further inquiries can be directed to the corresponding author.

## References

[1] Girschick HJ Raab P Surbaum S Trusen A Kirschner S Schneider P Chronic non-bacterial osteomyelitis in children. Ann Rheum Dis. (2005) 64(2):279–85. 10.1136/ard.2004.023838 15647436 PMC1755336

[2] Kumar TKJ Salim J Shamsudeen TJ. Chronic recurrent multifocal osteomyelitis - a rare clinical presentation and review of literature. J Orthop Case Reports. (2018) 8(3):3–6. 10.13107/jocr.2250-0685.1082 30584505 PMC6298712

[3] Hedrich CM Morbach H Reiser C Girschick HJ. New insights into adult and paediatric chronic non-bacterial osteomyelitis Cno. Curr Rheumatol Rep. (2020) 22(9):52. 10.1007/s11926-020-00928-1 32705386 PMC7378119

[4] Zhao Y Ferguson PJ. Chronic nonbacterial osteomyelitis and chronic recurrent multifocal osteomyelitis in children. Pediatr Clin N Am. (2018) 65(4):783–800. 10.1016/j.pcl.2018.04.003 30031498

[5] Bhat CS Anderson C Harbinson A McCann LJ Roderick M Finn A Chronic non bacterial osteitis- a multicentre study. Pediatr Rheumatol Online J. (2018) 16(1):74. 10.1186/s12969-018-0290-5 30466444 PMC6251121

[6] Kaut S Van den Wyngaert I Christiaens D Wouters C Noppe N Herregods N Chronic nonbacterial osteomyelitis in children: a multicentre Belgian cohort of 30 children. Pediatr Rheumatol Online J. (2022) 20(1):41. 10.1186/s12969-022-00698-3 35698069 PMC9195463

[7] Giedion A Holthusen W Masel LF Vischer D. Subacute and chronic “symmetrical” osteomyelitis. Ann Radiol Paris. (1972) 15(3):329–42. PMID: 4403064 4403064

[8] Jansson A Renner ED Ramser J Mayer A Haban M Meindl A Classification of non-bacterial osteitis: retrospective study of clinical, immunological and genetic aspects in 89 patients. Rheumatology. (2007) 46(1):154–60. 10.1093/rheumatology/kel190 16782988

[9] Rodriguez-Galindo C Allen CE. Langerhans cell histiocytosis. Blood. (2020) 135(16):1319–31. 10.1182/blood.2019000934 32106306

[10] Hospach T Langendoerfer M von Kalle T Maier J Dannecker GE. Spinal involvement in chronic recurrent multifocal osteomyelitis (Crmo) in childhood and effect of pamidronate. Eur J Pediatr. (2010) 169(9):1105–11. 10.1007/s00431-010-1188-5 20339868

[11] Jansson AF Grote V. Nonbacterial osteitis in children: data of a German incidence surveillance study. Acta Paediatrica. (2011) 100(8):1150–7. 10.1111/j.1651-2227.2011.02205.x 21352353

[12] Sułko J Ebisz M Bień S Błażkiewicz M Jurczyk M Namyślak M. Treatment of chronic recurrent multifocal osteomyelitis with bisphosphonates in children. Joint Bone Spine. (2019) 86(6):783–8. 10.1016/j.jbspin.2019.06.005 31216487

[13] Ma L Liu H Tang H Zhang Z Zou L Yu H Clinical characteristics and outcomes of chronic nonbacterial osteomyelitis in children: a multicenter case series. Pediatr Rheumatol Online J. (2022) 20(1):1. 10.1186/s12969-021-00657-4 34980193 PMC8722093

[14] Koneru S Magid MS Fritz J. Case of the season: asymmetric chronic recurrent multifocal osteomyelitis. Semin Roentgenol. (2022) 57(3):184–90. 10.1053/j.ro.2021.12.004 35842240

[15] Batheja D Munigangaiah S Jayanna HH Ghodke A. Contiguous three-level vertebral collapse in thoracic spine: a novel presentation of chronic recurrent multifocal osteomyelitis in 12 years old and review of literature. J Orthop Case Reports. (2021) 11(6):57–62. 10.13107/jocr.2021.v11.i06.2258 35437496 PMC9009478

[16] Yamashita K Calderaro C Labianca L Gajaseni P Weinstein SL. Chronic recurrent multifocal osteomyelitis (Crmo) involving spine: a case report and literature review. J Orthop Sci. (2021) 26(2):300–5. 10.1016/j.jos.2018.06.015 30153963

[17] Galeotti C Tatencloux S Adamsbaum C Koné-Paut I. Value of whole-body mri in vertebral fractures. Arch Pediatr. (2015) 22(3):279–82. 10.1016/j.arcped.2014.11.021. 25650082

[18] Hong CW Hsiao EC Horvai AE Link TM. Chronic recurrent multifocal osteomyelitis with an atypical presentation in an adult man. Skeletal Radiol. (2015) 44(9):1359–64. 10.1007/s00256-015-2130-8 25771734

[19] Habibi S Thompson E Thyagarajan MS Ramanan AV. Unusual presentation of spinal involvement in a child with chronic recurrent multifocal osteomyelitis. Int J Rheum Dis. (2013) 16(4):477–9. 10.1111/1756-185X.12102 23992272

[20] Deogaonkar K Ghandour A Jones A Ahuja S Lyons K. Chronic recurrent multifocal osteomyelitis presenting as acute scoliosis: a case report and review of literature. Eur Spine J. (2008) 17(Suppl 2):S248–52. 10.1007/s00586-007-0516-6 17912555 PMC2525918

[21] Walls T Bate J Moshal K. Vertebral collapse in an 8-year-old girl. J Paediatr Child Health. (2006) 42(4):212–4. 10.1111/j.1440-1754.2006.00832.x 16630324

[22] Chun CS. Chronic recurrent multifocal osteomyelitis of the spine and mandible: case report and review of the literature. Pediatrics. (2004) 113(4):e380–4. 10.1542/peds.113.4.e380 15060273

[23] Anderson SE Heini P Sauvain MJ Stauffer E Geiger L Johnston JO Imaging of chronic recurrent multifocal osteomyelitis of childhood first presenting with isolated primary spinal involvement. Skeletal Radiol. (2003) 32(6):328–36. 10.1007/s00256-002-0602-0 12761599

[24] Koné-Paut I Mannes I Dusser P. Chronic recurrent multifocal osteomyelitis (Crmo) and Juvenile Spondyloarthritis (Jspa): to what extent are they related? J Clin Med. (2023) 12(2):453. 10.3390/jcm12020453 PMC9867437 36675382

[25] Maksymowych WP. Bisphosphonates for arthritis–a confusing rationale. J Rheumatol. (2003) 30(3):430–4. PMID: 12610803 12610795

[26] Kostik MM Kopchak OL Chikova IA Isupova EA Mushkin AY. Comparison of different treatment approaches of pediatric chronic non-bacterial osteomyelitis. Rheumatol Int. (2019) 39(1):89–96. 10.1007/s00296-018-4151-9 30171342

[27] Gleeson H Wiltshire E Briody J Hall J Chaitow J Sillence D Childhood chronic recurrent multifocal osteomyelitis: pamidronate therapy decreases pain and improves vertebral shape. J Rheumatol. (2008) 35(4):707–12. PMID: 18381777 18381777

[28] Hofmann C Wurm M Schwarz T Neubauer H Beer M Girschick H A standardized clinical and radiological follow-up of patients with chronic non-bacterial osteomyelitis treated with pamidronate. Clin Exp Rheumatol. (2014) 32(4):604–9. PMID: 25065777 25065777

[29] Hofmann SR Kapplusch F Mäbert K Hedrich CM. The molecular pathophysiology of chronic non-bacterial osteomyelitis (Cno)-a systematic review. Mol Cell Pediatr. (2017) 4(1):7. 10.1186/s40348-017-0073-y 28685269 PMC5500598

[30] Schnabel A Nashawi M Anderson C Felsenstein S Lamoudi M Poole-Cowley J Tnf-Inhibitors or bisphosphonates in chronic nonbacterial osteomyelitis? - results of an international retrospective multicenter study. Clin Immunol. (2022) 238:109018. 10.1016/j.clim.2022.109018 35460903

[31] Zhao Y Wu EY Oliver MS Cooper AM Basiaga ML Vora SS Consensus treatment plans for chronic nonbacterial osteomyelitis refractory to nonsteroidal antiinflammatory drugs and/or with active spinal lesions. Arthritis Care Res. (2018) 70(8):1228–37. 10.1002/acr.23462 PMC5938153 29112802

[32] Zhao M Wu D Yu K Shen M. Clinical and genetic features of Chinese adult patients with chronic non-bacterial osteomyelitis: a single center report. Front Immunol. (2022) 13:860646. 10.3389/fimmu.2022.860646 35422809 PMC9002012

[33] Uehara N Kukita A Kyumoto-Nakamura Y Yamaza T Yasuda H Kukita T. Osteoblast-derived laminin-332 is a novel negative regulator of osteoclastogenesis in bone microenvironments. Lab Invest. (2017) 97(10):1235–44. 10.1038/labinvest.2017.55 28581488

