# Supplementary material for: Case report: Child chronic nonbacterial osteomyelitis with rapid progressive scoliosis-an association with disease?
Source: Front Pediatr. 2023 Mar 21;11:1076443. doi: 10.3389/fped.2023.1076443 (PMC10070962; doi:10.3389/fped.2023.1076443)
Supplement: Supplementary file 1 [file Datasheet1.pdf]

Supplementary Material

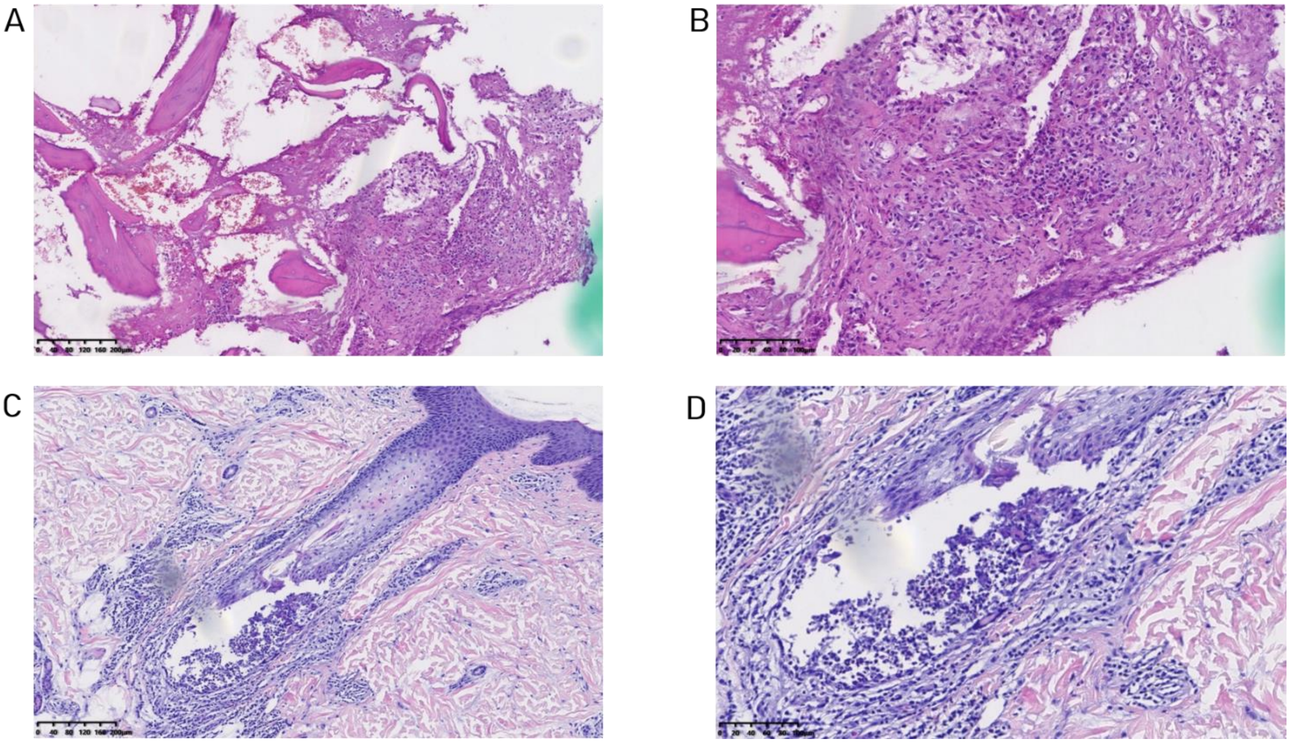

Supplementary Figure 1. T8 vertebral body tissue shows bone, cartilage and bone marrow with focal fibrous tissue hyperplasia with light microscope, HE 100× (A) and 200× (B). Skin tissue on the inner calf shows inflammatory cell infiltration, focal acute folliculitis, small pustule formation with light microscope, HE 100× (C) and 200× (D).

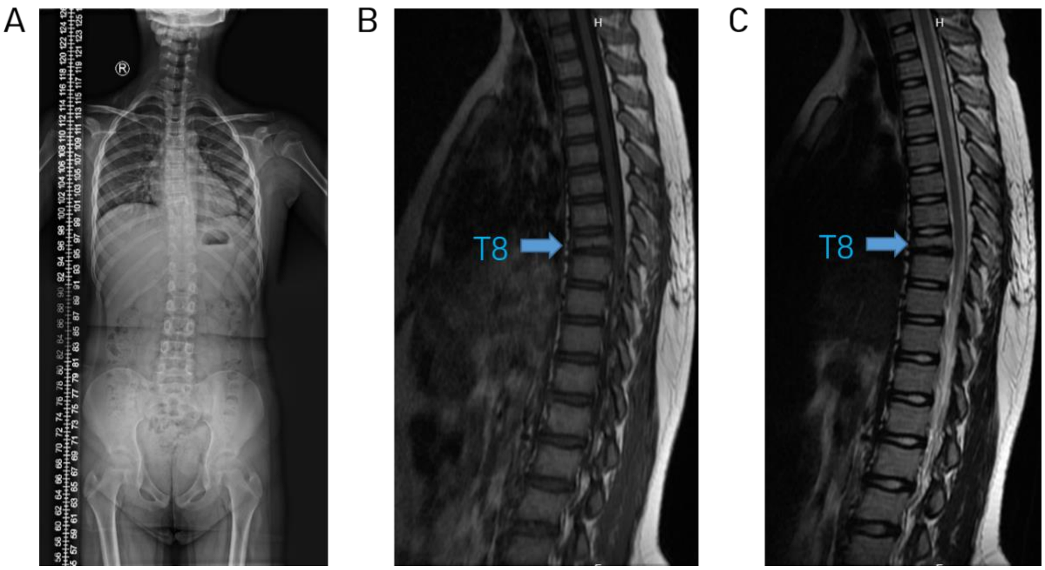

Supplementary Figure 2. Imaging changes after three months of therapy. (A) Standing spinal X-ray. (B) T1-weighted and (C) T2-weighted MRI images showed that T8 vertebra remained stable without progression (arrow).

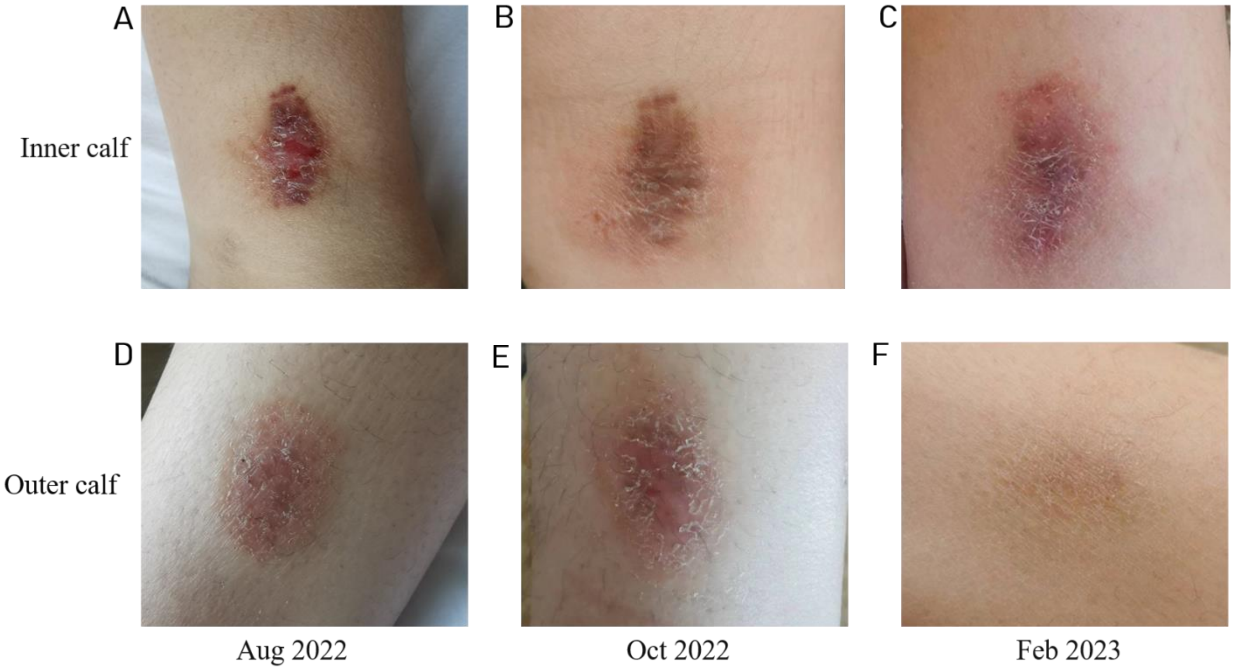

Supplementary Figure 3. Change in lesions on both sides of the calf before and after treatment. (A) Inner calf before treatment. (B) Inner calf after 3 months of treatment. (C) Inner calf after 6 months of treatment. (D) Outer calf before treatment. (E) Outer calf after 3 months of treatment. (F) Outer calf after 6 months of treatment.

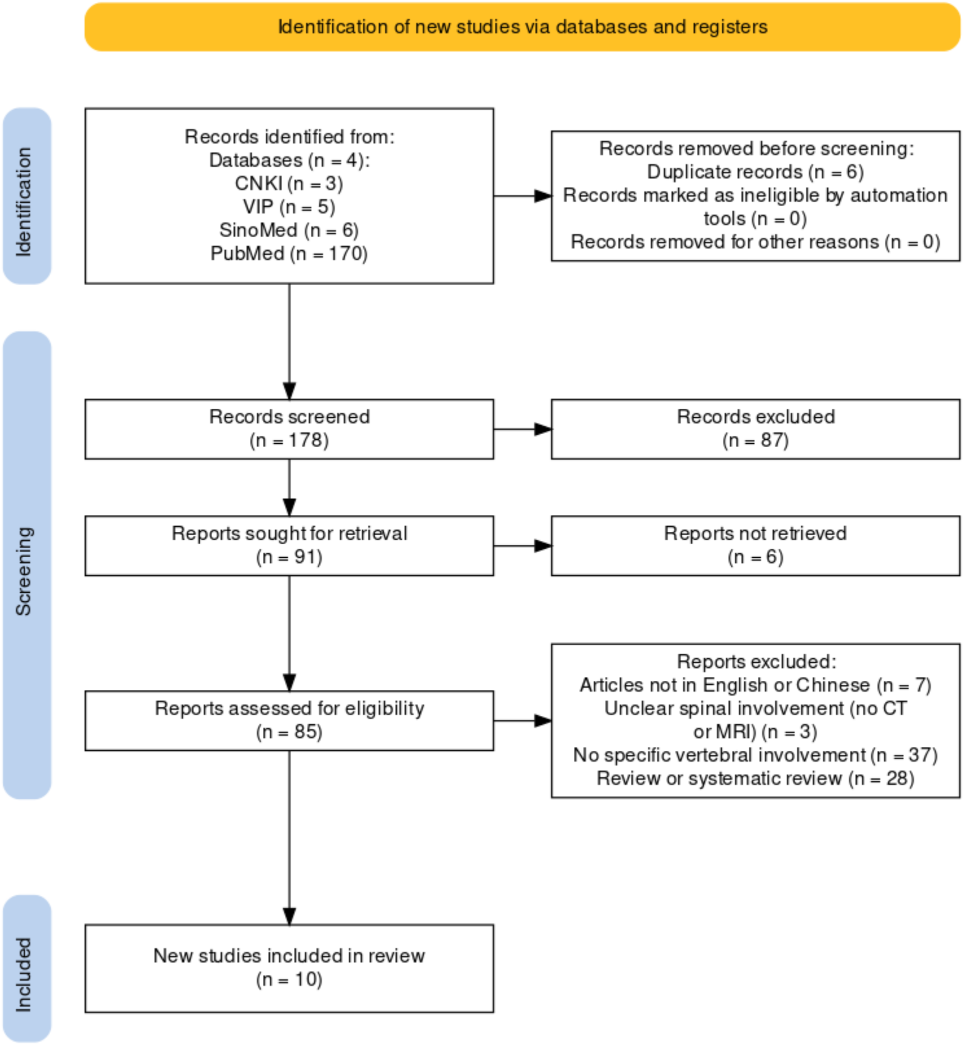

Supplementary Figure 4. PRISMA (2020) flow chart of the literature screening process.
